# First Report of Coexistence of *bla*_SFO–1_ and *bla*_NDM–1_ β-Lactamase Genes as Well as Colistin Resistance Gene *mcr-9* in a Transferrable Plasmid of a Clinical Isolate of *Enterobacter hormaechei*

**DOI:** 10.3389/fmicb.2021.676113

**Published:** 2021-06-18

**Authors:** Wenxiu Ai, Ying Zhou, Bingjie Wang, Qing Zhan, Longhua Hu, Yanlei Xu, Yinjuan Guo, Liangxing Wang, Fangyou Yu, Xiaolong Li

**Affiliations:** ^1^Department of Respiratory Medicine, The First Affiliated Hospital of Wenzhou Medical University, Wenzhou, China; ^2^Department of Clinical Laboratory Medicine, Shanghai Pulmonary Hospital, Tongji University School of Medicine, Shanghai, China; ^3^Shanghai Key Laboratory of Tuberculosis, Shanghai Pulmonary Hospital, Tongji University School of Medicine, Shanghai, China; ^4^Jiangxi Provincial Key Laboratory of Medicine, Clinical Laboratory of the Second Affiliated Hospital of Nanchang University, Nanchang, China; ^5^Department of Clinical Laboratory, The First Affiliated Hospital of Wenzhou Medical University, Wenzhou, China

**Keywords:** *Enterobacter hormaechei*, plasmid, *bla*_SFO–1_, *bla*_NDM–1_, *mcr-9*, IncHI2, WGS, mobile elements

## Abstract

Many antimicrobial resistance genes usually located on transferable plasmids are responsible for multiple antimicrobial resistance among multidrug-resistant (MDR) Gram-negative bacteria. The aim of this study is to characterize a carbapenemase-producing *Enterobacter hormaechei* 1575 isolate from the blood sample in a tertiary hospital in Wuhan, Hubei Province, China. Antimicrobial susceptibility test showed that 1575 was an MDR isolate. The whole genome sequencing (WGS) and comparative genomics were used to deeply analyze the molecular information of the 1575 and to explore the location and structure of antibiotic resistance genes. The three key resistance genes (*bla*_SFO–1_, *bla*_NDM–1_, and *mcr-9*) were verified by PCR, and the amplicons were subsequently sequenced. Moreover, the conjugation assay was also performed to determine the transferability of those resistance genes. Plasmid files were determined by the S1 nuclease pulsed-field gel electrophoresis (S1-PFGE). WGS revealed that p1575-1 plasmid was a conjugative plasmid that possessed the rare coexistence of *bla*_SFO–1_, *bla*_NDM–1_, and *mcr-9* genes and complete conjugative systems. And p1575-1 belonged to the plasmid incompatibility group IncHI2 and multilocus sequence typing ST102. Meanwhile, the pMLST type of p1575-1 was IncHI2-ST1. Conjugation assay proved that the MDR p1575-1 plasmid could be transferred to other recipients. S1-PFGE confirmed the location of plasmid with molecular weight of 342,447 bp. All these three resistant genes were flanked by various mobile elements, indicating that the *bla*_SFO–1_, *bla*_NDM–1_, and *mcr-9* could be transferred not only by the p1575-1 plasmid but also by these mobile elements. Taken together, we report for the first time the coexistence of *bla*_SFO–1_, *bla*_NDM–1_, and *mcr-9* on a transferable plasmid in a MDR clinical isolate *E. hormaechei*, which indicates the possibility of horizontal transfer of antibiotic resistance genes.

## Introduction

Carbapenem-resistant Enterobacteriaceae (CRE) has recently emerged as a serious threat to modern healthcare, challenging our present antibiotic treatment strategy (Chen et al., 2014). Moreover, the carbapenem-resistant and extended-spectrum β-lactamase (ESBL)-producing Enterobacteriaceae are also classified as the “priority pathogens” by the World Health Organization in 2017 (WHO, 2017; Tacconelli et al., 2018). Among all these Enterobacteriaceae isolates, *Enterobacter hormaechei* is a notorious nosocomial pathogen contributing to various infections, such as bacteremia, endocarditis, and lower respiratory, urinary tract, and intra-abdominal infections (Xu et al., 2015).

Recently, reports about the coexistence of a rare ESBL gene *bla*_SFO–1_ and carbapenemase genes were increased (Zhou et al., 2020). New Delhi metallo-lactamase (NDM-1), a β-lactam hydrolase, constitutes a critical and growingly important medical issue, since its resistance trait compromises the efficacy of almost all lactams (except aztreonam), including carbapenems (Dortet et al., 2014). Compared with other broad-spectrum β-lactamase, the *bla*_SFO–1_ gene is a low-incidence antimicrobial resistance gene and usually not subject to systematic monitoring, which puts it at risk of being missed (Zhao et al., 2015; Zhou et al., 2020). With the increase of infections caused by carbapenemase-producing bacteria and the lack of novel antibiotics (Gurjar, 2015), polymyxins have become the last-resort therapies in the treatment of infections caused by this kind of multidrug-resistant (MDR) bacterial (Olaitan et al., 2014; Yilmaz et al., 2016). Thus, once the strains are resistant to both carbapenems and polymyxins, the treatment will be very tough. The first plasmid-mediated colistin resistance gene *mcr-1* was identified in China from the plasmid of *Escherichia coli* and *Klebsiella pneumoniae* in IncI2 (Liu et al., 2016). *mcr-1* remains the main plasmid-mediated myxobacteria resistance gene, but *mcr-2* to *mcr-8* has been identified in different species in humans and animals (Wang et al., 2018; Nang et al., 2019). *mcr-9* has also been identified in Swedish ESBL isolates, including *Enterobacter cloacae*, *E. coli*, *Klebsiella acidophilus*, and *Citrobacter freundii* (Börjesson et al., 2020). Of particular concern is the spread of *mcr* genes into CRE, which would create strains that are potentially pan-drug resistant (PDR). So mobile colistin-resistant genes (*mcr*) have become an increasing public health concern.

It is common for the coexistence of mcr-9 with carbapenemases, such as *bla*_NDM–1_, *bla*_VIM–4_, and *bla*_IMP–4_ (Chavda et al., 2019; Chen et al., 2020). However, in this study, we found a clinical isolate of carbapenem-resistant *E. hormaechei*, which possessed the rare coexistence of *bla*_SFO–1_, *bla*_NDM–1_, and *mcr-9* genes. And we also explored the molecular basis for antibiotic resistance of this strain.

## Materials and Methods

### Bacterial Isolation and Identification

The *E. hormaechei* 1575 was isolated from the blood sample in a tertiary hospital in Wuhan, Hubei Province, China. The cultured bacteria were stored in glycerol broth at 80°C. And then samples were cultured on Colombian blood Agar plate and identified by matrix-assisted laser desorption/ionization time of flight mass spectrometry (MALDI-TOF MS) according to the manufacturer’s instructions and also by whole genome sequencing (WGS) (discussed below). *Escherichia coli* American Type Culture Collection (ATCC) 25922 was used as control strains for the identification of the species.

### Antimicrobial Susceptibility Testing

A total of 17 antimicrobial agents were tested, including imipenem (Ipm), meropenem (Mer), piperacillin–tazobactam (P/T), ceftazidime–avibactam (Caz/Avi), aztreonam (Azt), cefoxitin (Fox), cefotaxime (Ctx), cefepime (Cpe), ceftazidime (Caz), gentamicin (Gen), amikacin (AMK), ciprofloxacin (Cip), sulfamethoxazole (CoSMZ), tetracycline (Te), minocycline (Min), tigecycline (TGC), and polymyxin B (PB). The minimum inhibitory concentrations (MICs) of antimicrobial agents for the bacteria tested were determined using the broth microdilution method, and the susceptibility breakpoints were interpreted in accordance with the Clinical and Laboratory Standards Institute (CLSI) guideline (Clinical and Laboratory Standards Institute (CLSI), 2020), except for tigecycline and colistin, for which we used the European Committee on Antimicrobial Susceptibility Testing (EUCAST) breakpoints (Clinical and Laboratory Standards Institute (CLSI), 2020; EUCAST, 2020). AST was repeated three times in our study. *E. coli* ATCC 25922 was used as a control strain for the AST.

### Carbapenemase Phenotype Confirmation Testing

The modified carbapenem inactivation test (mCIM) was performed, according to CLSI 2020 standards [Clinical and Laboratory Standards Institute (CLSI), 2020], to verify carbapenemase production by the isolate. The tested strain 1575 was incubated with a meropenem disk (10 μg, OXOID, United Kingdom) immersed in the 2 ml of TSB suspension at 37°C for 4 h. *E. coli* ATCC 25922 was used as an indicator and with its 0.5 McFarland suspension uniformly coated on the Mueller Hinton Agar (MHA) plate. After the plate was dried for 3–10 min, the meropenem disk was removed from the suspension, and the excess medium was squeezed out. It was then placed on the MHA plate and incubated at 37°C for 18–24 h.

### Whole Genome Sequencing and Bioinformatics Analysis

Bacterial genomic DNA was isolated using the UltraClean Microbial Kit (Qiagen, NW, Germany) and sequenced from a sheared DNA library with average size of 15 kb (ranged from 10 to 20 kb) on a PacBio RSII sequencer (Pacific Biosciences, CA, United States), as well as a paired-end library with an average insert size of 350 bp (ranged from 150 to 600 kb) on a HiSeq sequencer (Illumina, CA, United States). Sequencing libraries were constructed using the NEBNext^®^ Ultra^TM^ II DNA Library Prep Kit for Illumina^®^ (second-generation sequencing) and the SMRTbell^®^ Express Template Prep Kit 2.0 kit (third-generation sequencing) and then loaded onto NovaSeq S4 flowcell and SMRT Cell 8 M DNA sequencing chip, respectively. The paired-end short Illumina reads were used to correct the long PacBio reads utilizing proovread (Hackl et al., 2014), and then the corrected PacBio reads were assembled *de novo* utilizing SMARTdenovo^1^. Antimicrobial resistance genes were identified by ResFinder 3.2 available at Center for Genomic Epidemiology^2^. The plasmid incompatibility groups, pMLST, and multilocus sequence typing (MLST) were identified by PlasmidFinder 2.1^3^, pMLST 2.0^4^, and MLST 2.0 software^5^, respectively. To verify whether the plasmid was also a conjugative plasmid, we used the OriT Finder website^6^ to conduct a detailed analysis of the conjugation module. The IS elements can be directly determined from the known website^7^. We used blast^8^ to determine similar plasmids by comparing their coverages and identities. The circular representation of p1575 was generated with CGview^9^. The plasmid linear graph was analyzed by Easyfig software^10^.

### PCR Amplifications and Sequencing

The isolate was verified for the presence of *bla*_SFO–1_-positive strains using PCR with the primers *bla*_SFO–1_-forward and *bla*_SFO–1_-reverse. Meanwhile, the other carbapenemase genes responsible for carbapenem resistance (*bla*_KPC_, *bla*_VIM_, *bla*_GES_, *bla*_IMP_, *bla*_SPM_, *bla*_OXA–23_, *bla*_OXA–48_, *bla*_SME_, *bla*_SIM_, and *bla*_NDM_) (Queenan and Bush, 2007; Nordmann et al., 2011) and the colistin resistance gene *mcr-9* were also detected by PCR. The DNA fragments were analyzed using gel electrophoresis on 1% agarose gels, and the amplicons were subsequently sequenced on both strands by TSINGKE sequencing (Table 1).

**TABLE 1:** Primers used in this study.

Amplicon	Product size (bp)	Temperature (°C)	Primer (5′–3′)	
			Forward	Reverse
*bla*_SFO–1_	796	53	TTCTGCTGTGGCTGAGTG	TGATGGTCGCTACGGTTAT
*mcr-9*	730	50.3	TTCCCTTTGTTCTGGTTG	TACTCGGTGCGATTCATA

### Conjugation Experiment

The horizontal transferability of *bla*_SFO–1_, *bla*_NDM–1_, and *mcr-9* was examined using conjugation assay. The *E. hormaechei* 1575 was used as donor strain, and the *E. coli* EC600 (rifampicin-resistant) was used as the recipient strain. The donors and recipients were cultured to the logarithmic phase (OD600 = 0.4–0.6), mixed in a 1:1 ratio, centrifuged at 8,000 *g* for 1 min, and resuspended them in 20 μl of Luria Bertani (LB) broth. The resuspension was spotted on the LB plates and incubated overnight at 37°C. The spots were then transferred to 15-ml centrifuge tubes and washed with 3 ml of LB broth. Subsequently, the serial dilutions were plated onto MH agar plates containing cefotaxime (8 μg/ml) and rifampicin (200 μg/ml). The donor cells and recipient cells were used separately as controls to ensure the effectiveness of the screening plate antibiotics. All transconjugants were confirmed by PCR for the presence of *bla*_SFO–1_, *bla*_NDM–1_, and *mcr-9* genes. Transconjugants were subjected to susceptibility assays. The conjugation frequency was calculated as the number of transconjugants per donor cell.

### S1 Pulsed-Field Gel Electrophoresis

S1 pulsed-field gel electrophoresis (S1-PFGE) was performed to obtain plasmid profiles in donor strains, recipient strains, and transconjugants, as described previously (Chen et al., 2011). Briefly, the isolates were embedded in 10 g/L of Seakem Gold gel, digested with endonuclease S1 nuclease (Takara, Dalian, China), and subjected to pulsed-field gel electrophoresis (parameters: 14°C, voltage 6 V/cm, electric field angle 120°, conversion time 4.0–40 s, and electrophoresis 19 h). The genomic DNA of *Salmonella enterica* serovar Braenderup H9812 strain cut with *Xba*I was used as a control standard strain and a molecular size marker.

### Nucleotide Accession Number

The complete nucleotide sequences of the chromosome of 1575, p1575-1, and p1575-2 were deposited as GenBank accession numbers CP068287, CP068288, and CP068289, respectively.

## Results

### *Enterobacter hormaechei* 1575 Was a Multidrug-Resistant Strain and Produced Carbapenemase

To clarify the antibiotic-resistant phenotype of *E. hormaechei* 1575, we tested the susceptibility of 17 antibiotics in this strain. As the results showed (Table 2), *E. hormaechei* 1575 was resistant to all β-lactam antibiotics (cephalosporins, carbapenems, penicillins, and monocyclic β-lactams), aminoglycosides, quinolones, and tetracycline. We found that 1575 was only susceptible to tigecycline, amikacin, and polymyxin B. Notably, for the ceftazidime–avibactam, a novel carbapenemase inhibitor, this isolate also exhibited high-level resistance.

**TABLE 2:** Antimicrobial drug susceptibility profiles.

Drug class	Antibiotic	MIC (mg/L)/antimicrobial susceptibility			
		1575	S/I/R	p1575-1-EC600	EC600
Carbapenems	Ipm	4	R	8	≤0.5
	Mer	>16	R	8	≤0.5
β-Lactam/β-lactamase	P/T	>128/4	R	64/4	≤4/4
Inhibitor complexes	Caz/Avi	>32/4	R	>32/4	≤0.25/4
Monocyclic β-lactam	Azt	>32	R	16	≤1
Cephalosporin	Fox	>32	R	16	4
	Ctx	>64	R	>64	≤1
	Cpe	>16	R	>16	≤0.5
	Caz	>32	R	>32	≤1
Fluoroquinolones	Cip	1	R	2	≤0.25
Folate metabolic pathway	CoSMZ	>2/38	-	>2/38	≤0.5/9.5
Inhibitors	Te	>16	R	>16	≤1
Tetracyclines	Min	>8	-	>8	≤2
	TGC	1	S	≤0.25	≤0.25
Polymyxin B	PB	2	S	2	≤0.5
Aminoglycosides	Gen	>16	R	>16	≤0.5
	AMK	≤2	S	≤2	≤2

*MIC, minimum inhibitory concentration; S, susceptible; R, resistant; I, intermediate; Ipm, imipenem; Mer, meropenem; P/T, piperacillin–tazobactam; Caz/Avi, ceftazidime–avibactam; Azt, aztreonam; Fox, cefoxitin; Ctx, cefotaxime; Cpe, cefepime; Caz, ceftazidime; Cip, ciprofloxacin; CoSMZ, sulfamethoxazole; Te, tetracycline; Min, minocycline; TGC, tigecycline; PB, polymyxin B; Gen, gentamicin; AMK, amikacin.*

Since *E. hormaechei* 1575 was resistant to both carbapenems and ceftazidime–avibactam, we used the mCIM assay to test preliminary whether this isolate produces carbapenemases. The result showed that *E. hormaechei* 1575 was positive for the mCIM assay, indicating that the isolate produced carbapenemases. Combining this strain with resistance to ceftazidime–avibactam, we speculated that *E. hormaechei* 1575 produced metallo-carbapenemase.

### *Enterobacter hormaechei* 1575 Co-harboring *bla*_SFO–1_, *bla*_NDM–1_, and *mcr-9* Resistance Genes

Through the resistance phenotype assays, we evaluated the clinical treatment challenges brought by this strain. Here, we continued to explore the associated molecular mechanism that contributed to such phenotype.

We used WGS to deeply mine the genomic information of the MDR bacteria. We found two plasmids in this isolate (named p1575-1 and p1575-2); and p1575-1 (CP068288) was larger with approximately 342,447 bp and sheltered multiple antibiotic resistance genes, especially including β-lactam resistance genes *bla*_SFO–1_, *bla*_NDM–1_, and colistin resistance gene *mcr-9* (Table 3). Besides, consistent with its multidrug resistance phenotype, p1575-1 also had multiple genes mediating resistance to quinolone (*qnrS1*), aminoglycosides [*aac(3)-IId*, *aph(3′)-Ia*, and *aph(6)-Id*], β-lactams (*bla*_TEM–1B_ and *bla*_LAP–2_), bleomycin *ble*_MBL_, trimethoprims (*dfrA14* and *dfrA19*), and MLS—macrolide [*mph(A)*] and tetracycline [*tet(D)*]. p1575-2 was a small plasmid of approximately 1,699 bp, with no resistance genes located on. We found the antibiotic-resistant plasmid by second-generation sequencing and further analyzed it by third-generation sequencing. Then we applied the PCR assay to verify these resistance genes. In addition, MLST analysis showed that *E. hormaechei* 1575 belonged to clone group ST102.

**TABLE 3:** General features, antimicrobial resistance genes, and mobile genetic elements of plasmids p1575-1 and p1575-2.

Location	Features	
	Size (bp)	Antimicrobial resistance genes
Chromosome	4,687,233	*bla*_ACT–5_, fosA
Plasmid-1	342,447	*bla*_SFO–1_, *mcr-9*, *bla*_NDM–1_, *bla*_TEM–1B_, *qnrS1*, *tet(D)*, *ble*_MBL_, *bla*_LAP–2_ *aac(3)-IId*, *aph(3*′*)-Ia*, *aph(6)-Id*, *mph(A)*, *dfrA14*, *dfrA19*
Plasmid-2	1,699	NA

*NA, not applicable.*

### Comparative Genomics of the Plasmid p1575-1 Carrying *bla*_SFO–1_, *bla*_NDM–1_, and *mcr-9*

**FIGURE 1:**
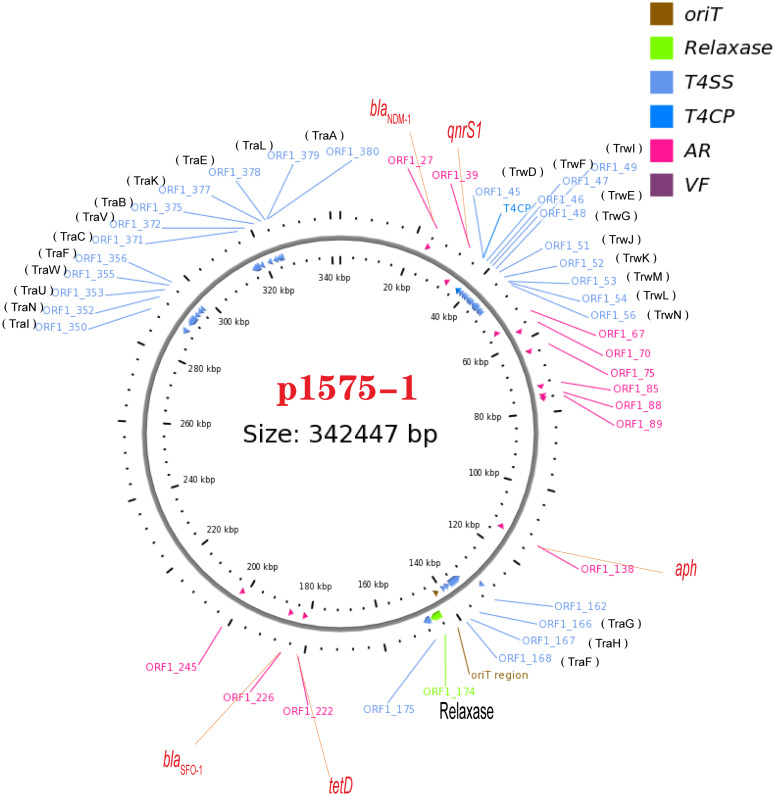
A conjugative plasmid p1575-1. AR (ARGs), acquired antibiotic resistance determinant genes; VF, virulence factors; ORF1-27, *bla*_NDM–1_; ORF1-39, *qnrS1*; ORF1-174, Relaxase; ORF1-222, *tetD*; ORF1-226, *bla*_SFO–1_.

We have known that the plasmid p1575-1 was the key plasmid for the contribution of the MDR phenotype; thus, exploring the characteristics of p1575-1 is the key to elucidating the spread of such bacteria and the mechanism of antibiotic resistance. p1575-1 is a 342,447-bp circular molecule with an average G + C content of 47.93% and was predicted to encode a total of 386 coding sequences (CDSs). Through the PlasmidFinder (see text footnote 3) and pMLST website (see text footnote 4), p1575-1 was typed as IncHI2 with Double Locus Sequence Type DLST1. To verify whether the p1575-1 plasmid was also a conjugative plasmid, we used the OriT Finder website (see text footnote 6) to conduct a detailed analysis of the conjugation module. Through the analysis, we identified the complete conjugative modules on the plasmid p1575-1, including the origin of transfer site (oriT), relaxase gene, gene encoding type IV coupling protein (T4CP), and gene cluster for bacterial type IV secretion system (T4SS) (Table 4). These results strongly suggested that p1575-1 is an MDR plasmid that can be transferred autonomously (Figure 1).

**TABLE 4:** Type IV secretion system components.

Type	P1575-1	
	Location	Gene/locus tag
OriT	141410–141661	-
Relaxase	143413–146559	ORF1_174
T4CP	36707–37702	ORF1_45
T4SS	36707–47032	ORF1_45ORF1_46 ORF1_47ORF1_48 ORF1_49 ORF1_50 ORF1_51 ORF1_52 ORF1_53 ORF1_54 ORF1_56
T4SS	130105–148643	ORF1_162 ORF1_166 ORF1_167 ORF1_168 ORF1_175
T4SS	287738–297020	ORF1_350 ORF1_352 ORF1_353 ORF1_355 ORF1_356
T4SS	315507–325118	ORF1_371 ORF1_372 ORF1_375 ORF1_377 ORF1_378 ORF1_379 ORF1_380

*T4CP, type IV coupling protein; T4SS, type IV secretion system; ORF, open reading frame.*

**FIGURE 2:**
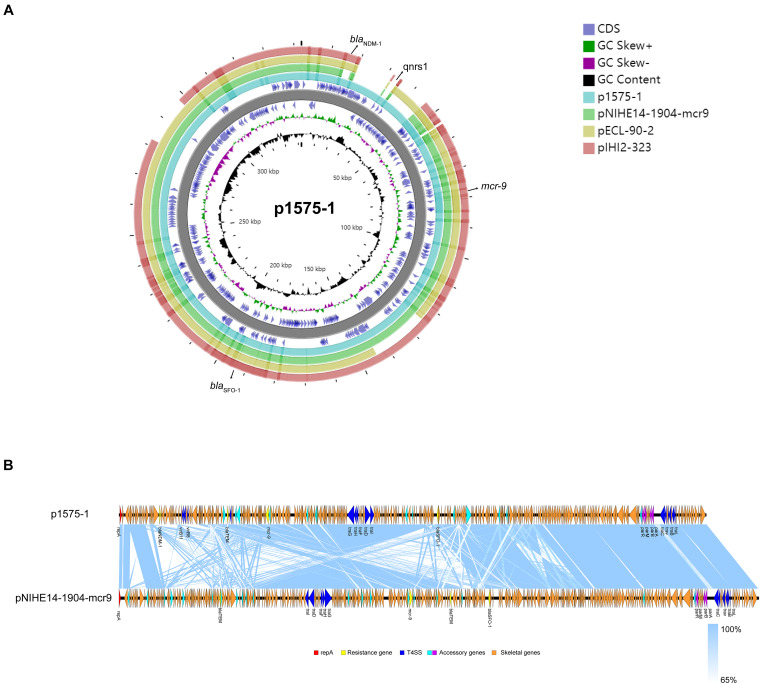
**(A)** Ring diagram representation of plasmid p1575-1. From the inside to the outside, the first circle represents the scale; the second circle represents GC content; the third circle represents the GC skew; the fourth and sixth circles represent the COG to which each CDS belongs; the fifth circle represents the backbone; the seventh to 10th circles represent p1575-1, pNIHE14-1904-mcr9, pECL-90-2, and pIHI2-323, respectively. GC, guanine + cytosine; *bla*_SFO–1_, extended-spectrum β-lactamases (ESBLs); *bla*_NDM–1_, New Delhi metallo-β-lactamase-1 gene; *mcr-9*, colistin resistance gene; *qnrs1*, fluoroquinolones gene. **(B)** Comparative analysis of the *mcr-9*-harboring plasmid characterized in this study with closely related plasmid pNIHE14-1904-*mcr9*. Open reading frames (ORFs) are portrayed by arrows and are depicted in different colors based on their predicted gene functions. The genes associated with the T4SS are indicated by dark blue arrows, while the genes involved in replication are indicated by red arrows. Resistance genes are indicated by yellow arrows, and accessory genes are indicated by light blue and purple arrows. Orange arrows represent the skeletal gene of the plasmid, and blue shading denotes shared regions of homology among different plasmids.

**FIGURE 3:**
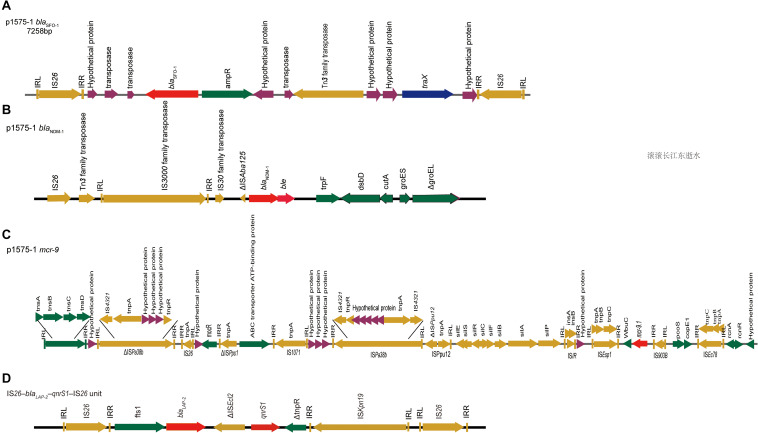
*bla*_SFO–1_, *bla*_NDM–1_, and *mcr-9* gene contigs. Genetic environments surrounding the *bla*_NDM–1_, *bla*_SFO–1_ and *mcr-9* genes in plasmid p1575-1. **(A)** The DNA fragments flanking the *bla*_SFO–1_ gene in plasmid p1575-1. **(B)** The DNA fragments flanking the *bla*_NDM–1_ gene in plasmid p1575-1. **(C)** The DNA fragments flanking the *mcr-9* gene in plasmid p1575-1. **(D)** The IS*26*–*bla*_LAP–2_–*qnrS1*–IS*26* module. Colored arrows indicate open reading frames, with dark green, dark yellow, dark blue, purple, and red arrows representing other genes, mobile and accessory elements, the individual conjugation-related genes, hypothetical proteins and transposases, and antibiotic resistance genes, respectively.

Moreover, we obtained three plasmids from the National Center for Biotechnology Information (NCBI) GenBank database for comparative analysis with our target plasmids. We found that p1575-1 had high homology with pNIHE14-1904-*mcr9* (GenBank accession no. LC570845.1) from *E. hormaechei*, with 91% query coverage and 99.97% sequence similarity. The other two plasmids held only 89% query coverage (pECL-90-2, CP061746.1) and 88% query coverage (pIHI2-323, CP049189.1) (Figure 2A). These results also suggested that these plasmids might have evolved from a single ancestor, or one might have evolved from the other. Finally, the pNIHE14-1904-*mcr9* was chosen as the reference plasmid for genome analysis, because of the high query coverage and sequence similarity (Figure 2B). The conjugative system of p1575-1 shared greater than 99% identity to that of pNIHE14-1904-*mcr9*, which in turn confirmed that the p1575-1 plasmid was a conjugative IncHI2 plasmid. Moreover, two IS*26* units were found on p1575-1. The first was the IS*26*–*bla*_SFO–1_–IS*26* transposable unit containing the SFO-1 ESBL gene (*bla*_SFO–1_) (Figure 3A). The other was the IS*26*–*bla*_LAP–2_–*qnrS1*–IS*26* module (Figure 3D) with two resistance genes included (*bla*_LAP–2_ and *qnrS1*). Remarkably, only p1575-1 plasmid harbored the second MDR gene IS*26* unit compared with other three IncHI2 plasmids.

Overall, these findings revealed that the p1575-1 plasmid was an MDR conjugative plasmid, which carried three key resistance genes (*bla*_SFO–1_, *bla*_NDM–1_, and *mcr-9*) and complete conjugative systems.

### p1575-1 Plasmid Could Transfer *bla*_SFO–1_, *bla*_NDM–1_, and *mcr-9* Genes

**FIGURE 4:**
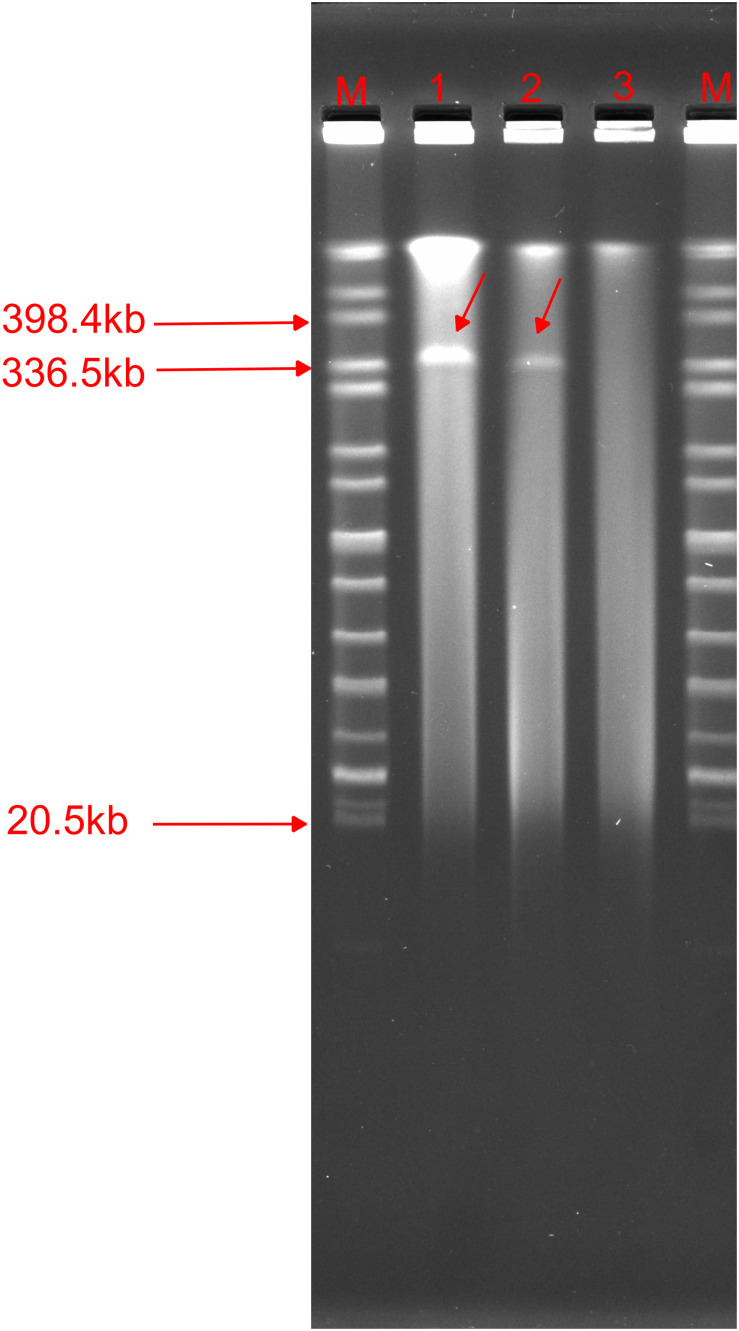
S1-nuclease pulsed-field gel electrophoresis profiles. M, *Salmonella enterica* serotype Braenderup strain H9812; 1,1575; 2, p1575-1-EC600; 3, EC600.

We found that the p1575-1 plasmid carried complete conjugative systems. Hence, we applied the conjugation assay to prove whether the MDR p1575-1 plasmid could infect other strains autonomously by conjugation. We identified that p1575-1 was able to be transferred to the rifampicin-resistant *Escherichia coli* EC600 *via* conjugation, p1575-1-EC600; and the conjugation frequency was estimated at (0.5–2) × 10^–6^ per donor cell. Then S1-PFGE revealed that *E. hormaechei* 1575 and p1575-1-EC600 contained the large plasmid (p1575-1) (336.5–398.4 kb) (Figure 4), consistent with the result of WGS. Besides, another plasmid (named p1575-2) was also found by WGS, a small plasmid approximately 1,699 bp, with no resistance genes on and could not be detected by S1-PFGE. Transconjugants were subjected to susceptibility assays. The antimicrobial susceptibility patterns are shown in Table 2. The transconjugants showed similar antibiotic susceptibility profile to the donor strain *E. hormaechei* 1575. The MICs of transconjugants were decreased compared with those of 1575, but they were both sensitive to tigecycline.

### Mobile Genetic Elements Associated With *bla*_SFO–1_, *bla*_NDM–1_, and *mcr-9*

Besides an in-depth analysis of the characteristics of MDR plasmids, we also analyzed the mobile elements flanking the resistant genes.

Our results showed that *bla*_SFO–1_ was located on a 7,258-bp IS*26* unit (IS*26–traX-ampR-bla*_SFO–1_*-*IS*26*) (Figure 3A). Genetic mapping of *bla*_SFO–1_ revealed that IS*26* and *ampR* were upstream and downstream of *bla*_SFO–1_, respectively. Tn*3* family, Tn*3* transposase DDE domain protein, and IncF plasmid conjugative transfer pilin acetylase, *traX*, were located downstream of *ampR*. Genetic mapping of *bla*_NDM–1_ revealed that the insertion sequence IS*3000* was interrupted by the insertion of a truncated ΔIS*Aba125* element. A bleomycin resistance gene, *ble*_MBL_, and dsbD, encoded oxidoreductase superfamily protein, were downstream of *bla*_NDM–1_ (Figure 3B). For *bla*_NDM–1_, a high similar genetic pattern was also observed in other plasmids, pNDM-BTR (McGann et al., 2015) (GenBank accession number KF534788, IncN1) and pNDM1-CBG (accession number CP046118, unpublished). In plasmid 1575-1, IS*5* family transposase (IS*903B*) was located upstream of *mcr-9.1*, whereas *wbuC*, IS*1R*, *sil*, *mocR*, IS*26*, and *tnsDCBA* were located downstream (Figure 3C). Besides, there were many other insertion sequences on the backbone where *mcr-9* was located. However, *qseB* and *qseC* regulatory genes were not found in association with the *mcr-9* gene.

## Discussion

The spread of *bla*NDM-1 among bacterial pathogens is of concern not only because of resistance to carbapenems but also because such pathogens typically are resistant to multiple antimicrobial drug classes, which leaves few treatment choices available (Kumarasamy et al., 2010; Moellering, 2010; Walsh, 2010). Not like the *bla*NDM-1, which receives widespread attention, the *bla*_SFO–1_ gene is not included in the routine surveillance, but it could be an effective weapon that various gram-negative bacteria could use to resist β-lactams (Matsumoto and Inoue, 1999); therefore, the prevalence of the coexistence of the *bla*_SFO–1_ gene and carbapenemase genes might be underestimated. Some studies reported the coexistence of *bla*_SFO–1_ and *bla*_NDM–1_ β-lactamase genes and fosfomycin resistance gene *fosA3* in *Escherichia coli* clinical isolate (Zhao et al., 2015) and the co-occurrence of *mcr-9* and *bla*_NDM–1_ in *Enterobacter cloacae* (Yuan et al., 2019; Faccone et al., 2020; Lin et al., 2020). However, in this study, we not only found the coexistence of *mcr-9* and *bla*_NDM–1_, but also a rare gene *bla*_SFO–1_ was detected on the same transferable plasmid. The presence of *bla*_SFO–1_ might confer resistance to more antibiotics. *mcr* is a family of genes found to promote colistin resistance in bacteria. As we all know, polymyxin antibiotic would be a good choice for *bla*_NDM–1_-positive strains, but we found *mcr-9* (Börjesson et al., 2020) in *E. hormaechei* 1575, which could reduce the sensitivity of the strain to polymyxin and increase its clinical menacing. Notably, the novel antibiotic ceftazidime–avibactam was also ineffective against 1575. Tigecycline is a last-resort antibiotic that is used to treat severe infections caused by extensively drug-resistant bacteria (Tasina et al., 2011) and may be used as a therapeutic drug for 1575. All these results indicated the *E. hormaechei* 1575 was MDR isolates and could only choose limited antibiotics. The presence of drug resistance genes strongly correlated with resistant phenotypes. The *E. hormaechei* 1575 was confirmed to produce carbapenemase. At the same time, two cases of MDR *E. cloacae* isolates had been reported to be ST102 in China (Cao et al., 2017), and this kind of strain was also found in our study. High attention should be given to its subsequent epidemic.

Previous studies showed that multiple resistance transfer of plasmids could result from rare gene capture events mediated by different mobile genetic elements, clustering, and combinatorial evolution of resistance genes and related mobile elements (Partridge and Tsafnat, 2018). Through the WGS and comparative genomics, we clarified that the key to mediating the antibiotic resistance of this strain was the p1575-1 resistant plasmid. The p1575-1 identified in this study was an IncHI2 conjugative plasmid, representing one of the most frequently encountered plasmid types in Enterobacteriaceae (Carattoli, 2009). Notably, IncHI2 plasmids are also broad-host-range, large (>250 kb) conjugative plasmids that mobilize metal and drug resistance genes within gram-negative pathogens (Bertrand et al., 2006; Novais et al., 2006; Roy Chowdhury et al., 2019). Meanwhile, IncHI2-ST1 plasmids always contributed to the dissemination of carbapenemase-encoding genes and are also reported frequently to play a critical role in the evolution of complex resistance phenotypes within disease-causing strains of Enterobacteriaceae (Roy Chowdhury et al., 2019). Moreover, IncHI2 plasmids contain the conjugal transfer gene regions *tra1* and *tra2*, likely contributing to the spread of resistance in the environment (Sherburne et al., 2000). In this study, we analyzed the conjugative modules of the p1575-1 plasmid and evaluated its mobility with conjugation assay. Like the classical IncHI2 plasmids, the p1575-1 plasmid held a complete conjugative system, and the conjugation frequencies ranged from 0.5 × 10^–6^ to 2 × 10^–6^ per donor cell. The IncHI2-type conjugative plasmids harboring *mcr-9* were also discovered previously, and the conjugation frequencies of those plasmid were 10^–4^ (Lin et al., 2020) or 2.03 × 10^–7^ (−5.42 × 10^–8^) (Cha et al., 2020), which were similar to our findings. Through the analysis, we identified the complete conjugative modules on the plasmid p1575-1, strongly suggesting that p1575-1 could be transferred autonomously. In addition to the conjugative plasmids, the capture, accumulation, and dissemination of resistance genes are largely due to the actions of mobile genetic elements, including insertion sequences, transposons, gene cassettes, and integrons. In this study, we found that all these three resistance genes were flanked by several mobile elements. The *bla*_SFO–1_ was located in an IS*26* composite transposon. IS*6* family elements IS*26* have played a pivotal role in the dissemination of resistance determinants in Gram-negative bacteria; thus, *bla*_SFO–1_ held the potential to transfer to other strains. AmpR, a class of DNA-binding regulatory protein, belongs to the LysR family of transcriptional regulators (Henikoff et al., 1988; Bartowsky and Normark, 1993). AmpR is confirmed to be a transcriptional activator in the presence of certain β-lactam antibiotics in the culture medium and a repressor in their absence (Lindberg et al., 1988). The presence of *ampR* seems to be a disadvantage for the host strain because *E. cloacae* become highly resistant to β-lactams (Matsumoto and Inoue, 1999). The movement of IS*26* is originally demonstrated to occur by replicative transposition. Moreover, the *bla*SFO-1 genes in previous identifications were located on non-conjugative plasmids (Guo et al., 2012). In our study, the conjugative *bla*_SFO–1_-*bla*_NDM–1_-*mcr-9*-bearing plasmid belonged to IncHI2, which is a kind of broad-host-range mobile plasmid and might greatly accelerate the dissemination of the *bla*_SFO–1_ genes. Previous reports showed that the *bla*_NDM–1_ genes in Enterobacteriaceae were usually on 50- to 200-kb plasmids belonging to IncL/M, IncHI1, IncFIIs, IncF, or untypable (Ahmad et al., 2018). IS*Aba125* and Tn*125* are always associated with the *bla*_NDM–1_ gene. Upstream of the *bla*_NDM–1_ gene, a truncated insertion sequence, IS*Aba125*, was identified, which provides a promoter for the expression of *bla*_NDM–1_ (Carattoli et al., 2012), and the presence of *ble* andΔ*tnpA* genes suggests a possible hypothesis that *bla*_NDM–1_ originates from *Acinetobacter baumannii* (Poirel et al., 2012; Toleman et al., 2012). Besides, phosphoribosylanthranilate isomerase gene *trpF* was identified in the downstream sequences of the *ble*_MBL_ gene (Liu et al., 2013). In addition, *qnrS1* in IS*26*–*bla*_LAP–2_–*qnrS1*–IS*26* unit (3D) was also found, consistent with our AST results. In the IncHI2 plasmid, the *mcr-9* allele always inserted an IS*903B* element and an IS*Esp1*, encoding a cupin fold metalloprotein, *wbuC* family (Yuan et al., 2019; Börjesson et al., 2020), which was consistent with our results. Because *mcr-9.1* was located between IS*903B* and IS*26*, these flanking sequences can also be potentially transferred to other bacteria along with *mcr-9.1*. All results indicated that the resistant plasmid carried by *E. hormaechei* 1575 can be spontaneously transmitted to other strains through conjugation, which had great potential to cause clinical epidemics. *qseB* and *qseC* regulatory genes were found in association with the *mcr-9* gene and played an important role in mediating polymyxin resistance (Chavda et al., 2019; Kieffer et al., 2019). The lack of two key regulators (*qseB* and *qseC*) may explain why *E. hormaechei* 1575 carrying *mcr-9* did not exhibit a high resistance level to colistin (MIC, 2 μg/ml). Serious importance needs to be taken on this phenomenon.

In this study, all the resistant genes located on the p1575-1 plasmid were found to be chimeric with multiple IS sequences and various mobile elements, indicating that *bla*_SFO–1_, *bla*_NDM–1_, and *mcr-9* could be transferred not only by the p1575-1 plasmid but also by these mobile genes.

## Conclusion

In this study, we report the coexistence of *bla*_SFO–1_, *bla*_NDM–1_, and *mcr-9* encoding one transferable IncHI2 plasmid in an *E. hormaechei* isolate. The co-occurrence of *bla*_SFO–1_, *bla*_NDM–1_, and *mcr-9* (as well as many associated resistance genes) caused *E. hormaechei* 1575 to be highly resistant not only to carbapenems but also to novel antibiotic ceftazidime–avibactam. At the same time, enough attention should be given to the dissemination of colistin resistance genes *mcr-9*, as polymyxin has been considered to be the “last-resort” antibiotic to treat human infections caused by CRE. Yet more worryingly, these genes are associated with various mobile elements and conjugative plasmids. The presence of multiple mobile elements indicates that horizontal gene transfer events play a key role in the acquisition of antibiotic resistance and the evolution of plasmids. Future studies are necessary to evaluate the prevalence of these plasmids among clinical isolates in China and other countries.

## Data Availability Statement

The datasets presented in this study can be found in online repositories. The names of the repository/repositories and accession number(s) can be found in the article/supplementary material.

## Ethics Statement

As the *E. hormaechei* clinical isolate in this study was part of the routine hospital laboratory procedure, the Ethics Committee of the Shanghai Pulmonary Hospital of Tongji University School of Medicine approved our study.

## Author Contributions

All authors contributed to data analysis and drafting or revising the article, gave the final approval of the version to be published, and agreed to be accountable for all aspects of the work.

## Conflict of Interest

The authors declare that the research was conducted in the absence of any commercial or financial relationships that could be construed as a potential conflict of interest.

## Footnotes

1
https://github.com/ruanjue/smartdenovo

2
https://cge.cbs.dtu.dk/services/ResFinder/

3
https://cge.cbs.dtu.dk/services/PlasmidFinder/

4
https://cge.cbs.dtu.dk/services/pMLST/

5
https://cge.cbs.dtu.dk/services/MLST/

6
https://bioinfo-mml.sjtu.edu.cn/oriTfinder/

7
https://www-is.biotoul.fr/

8
https://blast.ncbi.nlm.nih.gov/Blast.cgi

9
http://stothard.afns.ualberta.ca/cgview_server/

10
http://mjsull.github.io/Easyfig/
