# Corrigendum: First Report of Coexistence of *bla*_SFO-1_ and *bla*_NDM-1_ β-Lactamase Genes as Well as Colistin Resistance Gene *mcr-9* in a Transferrable Plasmid of a Clinical Isolate of *Enterobacter hormaechei*

**DOI:** 10.3389/fmicb.2021.741628

**Published:** 2021-09-28

**Authors:** Wenxiu Ai, Ying Zhou, Bingjie Wang, Qing Zhan, Longhua Hu, Yanlei Xu, Yinjuan Guo, Liangxing Wang, Fangyou Yu, Xiaolong Li

**Affiliations:** ^1^Department of Respiratory Medicine, The First Affiliated Hospital of Wenzhou Medical University, Wenzhou, China; ^2^Department of Clinical Laboratory Medicine, Shanghai Pulmonary Hospital, Tongji University School of Medicine, Shanghai, China; ^3^Shanghai Key Laboratory of Tuberculosis, Shanghai Pulmonary Hospital, Tongji University School of Medicine, Shanghai, China; ^4^Jiangxi Provincial Key Laboratory of Medicine, Clinical Laboratory of the Second Affiliated Hospital of Nanchang University, Nanchang, China; ^5^Department of Clinical Laboratory, The First Affiliated Hospital of Wenzhou Medical University, Wenzhou, China

**Keywords:** *Enterobacter hormaechei*, plasmid, *bla*_SFO−1_, *bla*_NDM−1_, *mcr-9*, IncHI2, WGS, mobile elements

In the original article, there were two errors in the main text. The sentence “The conjugation frequency was calculated as the number of transconjugants per recipient” should have read “The conjugation frequency was calculated as the number of transconjugants per donor cell.”

A correction has been made to **Materials and Methods, Conjugation Experiment**:

“The horizontal transferability of *bla*_SFO−1_, *bla*_NDM−1_, and *mcr-9* was examined using conjugation assay. The *E. hormaechei* 1575 was used as donor strain, and the *E. coli* EC600 (rifampicin-resistant) was used as the recipient strain. The donors and recipients were cultured to the logarithmic phase (OD600 = 0.4–0.6), mixed in a 1:1 ratio, centrifuged at 8,000 *g* for 1 min, and resuspended them in 20 μl of Luria Bertani (LB) broth. The resuspension was spotted on the LB plates and incubated overnight at 37°C. The spots were then transferred to 15-ml centrifuge tubes and washed with 3 ml of LB broth. Subsequently, the serial dilutions were plated onto MH agar plates containing cefotaxime (8 μg/ml) and rifampicin (200 μg/ml). The donor cells and recipient cells were used separately as controls to ensure the effectiveness of the screening plate antibiotics. All transconjugants were confirmed by PCR for the presence of *bla*_SFO−1_, *bla*_NDM−1_, and *mcr-9* genes. Transconjugants were subjected to susceptibility assays. The conjugation frequency was calculated as the number of transconjugants per donor cell.”

In addition, the sentence “The *bla*_SFO−1_ was located in an IS*26* integron” should have read “The *bla*_SFO−1_ was located in an IS*26* composite transposon.”

A correction has been made to **Discussion, second paragraph**:

“Previous studies showed that multiple resistance transfer of plasmids could result from rare gene capture events mediated by different mobile genetic elements, clustering, and combinatorial evolution of resistance genes and related mobile elements (Partridge and Tsafnat, 2018). Through the WGS and comparative genomics, we clarified that the key to mediating the antibiotic resistance of this strain was the p1575-1 resistant plasmid. The p1575-1 identified in this study was an IncHI2 conjugative plasmid, representing one of the most frequently encountered plasmid types in Enterobacteriaceae (Carattoli, 2009). Notably, IncHI2 plasmids are also broad-host-range, large (>250 kb) conjugative plasmids that mobilize metal and drug resistance genes within gram-negative pathogens (Bertrand et al., 2006; Novais et al., 2006; Roy Chowdhury et al., 2019). Meanwhile, IncHI2-ST1 plasmids always contributed to the dissemination of carbapenemase-encoding genes and are also reported frequently to play a critical role in the evolution of complex resistance phenotypes within disease-causing strains of Enterobacteriaceae (Roy Chowdhury et al., 2019). Moreover, IncHI2 plasmids contain the conjugal transfer gene regions *tra1* and *tra2*, likely contributing to the spread of resistance in the environment (Sherburne et al., 2000). In this study, we analyzed the conjugative modules of the p1575-1 plasmid and evaluated its mobility with conjugation assay. Like the classical IncHI2 plasmids, the p1575-1 plasmid held a complete conjugative system, and the conjugation frequencies ranged from 0.5 × 10^−6^ to 2 × 10^−6^ per donor cell. The IncHI2-type conjugative plasmids harboring *mcr-9* were also discovered previously, and the conjugation frequencies of those plasmid were 10^−4^ (Lin et al., 2020) or 2.03 × 10^−7^ (−5.42 × 10^−8^) (Cha et al., 2020), which were similar to our findings. Through the analysis, we identified the complete conjugative modules on the plasmid p1575-1, strongly suggesting that p1575-1 could be transferred autonomously. In addition to the conjugative plasmids, the capture, accumulation, and dissemination of resistance genes are largely due to the actions of mobile genetic elements, including insertion sequences, transposons, gene cassettes, and integrons. In this study, we found that all these three resistance genes were flanked by several mobile elements. The *bla*_SFO−1_ was located in an IS*26* composite transposon. IS*6* family elements IS*26* have played a pivotal role in the dissemination of resistance determinants in Gram-negative bacteria; thus, *bla*_SFO−1_ held the potential to transfer to other strains. AmpR, a class of DNA-binding regulatory protein, belongs to the LysR family of transcriptional regulators (Henikoff et al., 1988; Bartowsky and Normark, 1993). AmpR is confirmed to be a transcriptional activator in the presence of certain β-lactam antibiotics in the culture medium and a repressor in their absence (Lindberg et al., 1988). The presence of *ampR* seems to be a disadvantage for the host strain because *E. cloacae* become highly resistant to β-lactams (Matsumoto and Inoue, 1999). The movement of IS*26* is originally demonstrated to occur by replicative transposition. Moreover, the *bla*SFO-1 genes in previous identifications were located on non-conjugative plasmids (Guo et al., 2012). In our study, the conjugative *bla*_SFO−1_-*bla*_NDM−1_-*mcr-9*-bearing plasmid belonged to IncHI2, which is a kind of broad-host-range mobile plasmid and might greatly accelerate the dissemination of the *bla*_SFO−1_ genes. Previous reports showed that the *bla*_NDM−1_ genes in Enterobacteriaceae were usually on 50- to 200-kb plasmids belonging to IncL/M, IncHI1, IncFIIs, IncF, or untypable (Ahmad et al., 2018). IS*Aba125* and Tn*125* are always associated with the *bla*_NDM−1_ gene. Upstream of the *bla*_NDM−1_ gene, a truncated insertion sequence, IS*Aba125*, was identified, which provides a promoter for the expression of *bla*_NDM−1_ (Carattoli et al., 2012), and the presence of *ble* andΔ*tnpA* genes suggests a possible hypothesis that *bla*_NDM−1_ originates from *Acinetobacter baumannii* (Poirel et al., 2012; Toleman et al., 2012). Besides, phosphoribosylanthranilate isomerase gene *trpF* was identified in the downstream sequences of the *ble*_MBL_ gene (Liu et al., 2013). In addition, *qnrS1* in IS*26*–*bla*_LAP−2_-*qnrS1*–IS*26* unit (3D) was also found, consistent with our AST results. In the IncHI2 plasmid, the *mcr-9* allele always inserted an IS*903B* element and an IS*Esp1*, encoding a cupin fold metalloprotein, *wbuC* family (Yuan et al., 2019; Börjesson et al., 2020), which was consistent with our results. Because *mcr-9.1* was located between IS*903B* and IS*26*, these flanking sequences can also be potentially transferred to other bacteria along with *mcr-9.1*. All results indicated that the resistant plasmid carried by *E. hormaechei* 1575 can be spontaneously transmitted to other strains through conjugation, which had great potential to cause clinical epidemics. *qseB* and *qseC* regulatory genes were found in association with the *mcr-9* gene and played an important role in mediating polymyxin resistance (Chavda et al., 2019; Kieffer et al., 2019). The lack of two key regulators (*qseB* and *qseC*) may explain why *E. hormaechei* 1575 carrying *mcr-9* did not exhibit a high resistance level to colistin (MIC, 2 μg/ml). Serious importance needs to be taken on this phenomenon.”

The authors apologize for these errors and state that they do not change the scientific conclusions of the article in any way. The original article has been updated.

## Publisher's Note

All claims expressed in this article are solely those of the authors and do not necessarily represent those of their affiliated organizations, or those of the publisher, the editors and the reviewers. Any product that may be evaluated in this article, or claim that may be made by its manufacturer, is not guaranteed or endorsed by the publisher.
